# Implementation and maintenance of infant dietary diversity in Zimbabwe: contribution of food and water insecurity

**DOI:** 10.1186/s40795-022-00622-8

**Published:** 2022-11-18

**Authors:** Nadia Koyratty, Mduduzi N. N. Mbuya, Andrew D. Jones, Roseanne C. Schuster, Katarzyna Kordas, Chin-Shang Li, Naume V. Tavengwa, Florence D. Majo, Bernard Chasekwa, Robert Ntozini, Jean H. Humphrey, Laura E. Smith

**Affiliations:** 1 Poverty, Health and Nutrition Department, International Food Policy Research Institute Washington DC, Washington, DC USA; 2 Global Alliance for Improved Nutrition, Washington, DC 20036 USA; 3 Department of Nutritional Sciences, School of Public Health, University of Michigan, Ann Arbor, MI 48109 USA; 4 Center for Global Health, School of Human Evolution and Social Change, Arizona State University, Tempe, AZ 85281 USA; 5 School of Nursing, The State University of New York, University at Buffalo, Buffalo, NY 14214 USA; 6 Zvitambo Institute for Maternal and Child Health Research, Harare, Zimbabwe; 7 Department of International Health, Johns Hopkins Bloomberg School of Public Health, Baltimore, MD 21205 USA; 8 Department of Public and Ecosystem Health, Cornell University, Ithaca, NY 14853 USA

**Keywords:** Food security, Minimal dietary diversity, Food availability, Food quality, Food access

## Abstract

**Background:**

Inadequate food and water resources negatively affect child health and the efficiency of nutrition interventions.

**Methods:**

We used data from the SHINE trial to investigate the associations of food insecurity (FI) and water insecurity (WI) on mothers’ implementation and maintenance of minimum infant dietary diversity (MIDD). We conducted factor analysis to identify and score dimensions of FI (poor access, household shocks, low availability & quality), and WI (poor access, poor quality and low reliability). MIDD implementation (*n* = 636) was adequate if infants aged 12 months (M12) ate ≥ four food groups. MIDD maintenance (*n* = 624) was categorized into four mutually exclusive groups: A (unmet MIDD at both M12 and M18), B (unmet MIDD at M12 only), C (unmet MIDD at M18 only), and D (met MIDD at both M12 and M18). We used multivariable-adjusted binary logistic and multinomial regressions to determine likelihood of MIDD implementation, and of belonging to MIDD maintenance groups A-C (poor maintenance groups), compared to group D, respectively.

**Results:**

Low food availability & quality were negatively associated with implementation (OR = 0.81; 0.69, 0.97), and maintenance (OR_B_ = 1.29; 1.07, 1.56). Poor water quality was positively associated with implementation (OR = 1.25; 1.08, 1.44), but inconsistently associated with maintenance, with higher odds of infants being in group C (OR = 1.39; 1.08, 1.79), and lower odds of being in group B (OR = 0.80; 0.66, 0.96).

**Conclusion:**

Food security should be prioritized for adequate implementation and maintenance of infant diets during complementary feeding. The inconsistent findings with water quality indicate the need for further research on WI and infant feeding.

**Supplementary Information:**

The online version contains supplementary material available at 10.1186/s40795-022-00622-8.

## Introduction

Complementary feeding (CF) between 6–23 months is one of the most effective strategies to address chronic undernutrition such as stunting [1–3], which affects 21% of infants under five years old worldwide [4]. Indeed, most growth faltering occurs during this time interval, and is considered largely irreversible beyond two years of age [5]. Adequate nutrition during this early period of life is critical for child survival, growth, development and future achievement [6]. However, from six months onwards, breastmilk alone does not meet the growing infant’s caloric and nutrient needs [7]. The key practice then includes the gradual introduction of a diverse variety of age-appropriate foods with continued breastfeeding [8]. A study in 21 countries, including 12 in Africa, found that minimum infant dietary diversity (MIDD) is the CF indicator most consistently associated with positive growth patterns [9]. Although many countries have scaled up CF interventions [3], evidence from 80 low- and middle-income countries (LMICs) indicates that achieving MIDD remains a public health challenge [10]. Thus, inadequate CF may explain why nutrition education and/ or supplementation interventions have only modestly improved child growth [1, 11, 12]. There are several reasons for this: 1) infants have smaller stomachs and may not be able to ingest a lot in one feeding, thus requiring increased feeding frequencies; 2) mothers may not know what or how to feed the infants adequately, and; 3) socio-economic and environmental situations, social norms and cultural beliefs that hinder appropriate feeding practices. Therefore, advancing knowledge about how to improve implementation and maintenance of MIDD at large scale should be prioritized, especially among those who receive nutrition-specific interventions [13, 14].

The UNICEF framework for undernutrition describes some underlying factors that influence CF, and highlights that a multisectoral approach is required to address it [15]. An essential consideration that is often overlooked is the availability of resources that households need to implement adequate infant feeding, and to maintain those feeding practices over time [16]. Among the necessary household resources, adequate food and water may be transformative for the success of nutrition education and behavior change interventions because they significantly affect whether caregivers are able to implement and maintain recommended CF practices via various pathways [17–20]. Therefore, food insecurity (FI) and water insecurity (WI) represent significant risks to diet and nutrition adequacy of infants.

Food insecurity (FI) may affect infant diet through pathways such as food availability, affordability, access, quantity and/ or quality [21, 22]. Although food supplementation and provision interventions have improved child health [23–25], reports linking these interventions to CF are scarce [19, 26–28]. Additionally, follow-up after the end of nutrition interventions rarely ascertains how FI affects the long-term maintenance of recommended infant feeding practices. Existing evidence is primarily from cross-sectional studies that identify FI as a determinant of CF [27, 29–34]. Three studies have investigated this association longitudinally among mother-infant dyads in rural Bangladesh [33, 35], and in informal settlements in Kenya [36]. Lower FI was associated with improved CF, but only in Kenya was the study implemented within a nutrition intervention [36].

Until recently, the contribution of WI to undernutrition had only been considered through the sanitation and hygiene pathways, via access to clean and safe water. Its role in CF remains understudied [37], although various pathways exist to link them [38, 39]. First, water availability may affect CF because of its importance in food handling and preparation (e.g., cleaning raw produce, boiling, steaming, washing dishes and kitchen utensils). Since preparation of culture-specific complementary foods can be water-intensive (e.g., legumes, grains, nuts, vegetables), inadequate water quantity and quality may affect food choices and diet diversity [40, 41]. Second, poor water access limits the ability of women to prepare diverse foods and feed their children [40, 41], because water collection activities are time consuming and often physically challenging [42]. In India, infants from households with access to piped water within or close to the home were 2% more likely to meet MIDD, compared to infants from households that needed to fetch water elsewhere [37].

FI and WI often coexist in LMICs [17], including in Zimbabwe, where 38% of the rural population is food insecure (based on the coping strategy index), and a similar percentage is WI (based on WHO’s definition of access to improved water) [43, 44]. In the Sanitation Hygiene and Infant Nutrition Efficacy (SHINE) trial, 69% of all rural Zimbabwean infants whose mothers were randomized to a nutrition intervention received an adequately diverse diet at 12 months of age [45]. This was in comparison to 52% of infants from the control arm whose mothers did not receive the intervention (p < 0.01). It is unclear why the recommended MIDD was not implemented by all mothers in SHINE’s nutrition intervention arm. There is also no indication of whether the mothers’ feeding behavior changed after the end of the nutrition education intervention or at the end of the trial when the infants were 18 months old. Since SHINE’s nutrition intervention was a behavioral change intervention, distal factors such as household-level FI and WI may be influencing the infant feeding behaviors. To bridge the information gap on the links between FI and WI and MIDD, we hypothesized that higher FI and WI would be associated with lower MIDD implementation and maintenance among by caregivers who received CF education.

## Methodology

### Study design

The SHINE trial design and outcomes have been published previously [45]. Briefly, SHINE was a 4-arm cluster-randomized trial testing the independent and combined effects of infant and young child feeding (IYCF) and household water, sanitation, and hygiene (WASH) on child stunting and anemia. It was a community-based intervention implemented in rural areas of Shurugwi and Chirumanzu districts in Zimbabwe. The districts were divided into clusters, each defined as a catchment area serviced by 1–4 village health workers (VHW) of the Ministry of Health and Child Care. Between 22 November 2012 and 27 March 2015, pregnant women, 15–49 years old, who were permanent residents of those rural areas were enrolled. The infants born to these mothers were then followed over time to ascertain stunting prevalence. The analyses presented in this paper focus on the IYCF arm (*n* = 1148 live born infants). The IYCF intervention included six nutrition education modules delivered by VHW during 15 home visits to participating women. The intervention which promoted WHO recommended feeding practices, adapted to the local context, were delivered at monthly intervals starting at infant age 5 months (Table 1). From the 6-month home visit until the 18-month visit, a daily supply of 20 g of Nutributter® was provided to the caregiver to supplement the diet of the index infant. Additional detail on the IYCF protocol is available online. SHINE’s IYCF arm was chosen for our analyses for two reasons: 1) the children showed improvements in growth compared to the other SHINE arms [45], and 2) it allows the exploration of the effects of FI and WI on recommended feeding practices because nutrition education was targeted without direct intervention on food or water.

**Table 1:** Education modules in the Infant and Young Child Feeding (IYCF) Nutrition Intervention arm of the Sanitation Hygiene and Infant Nutrition Efficacy (SHINE) Trial

**Specific Module**	**Target delivery time**	**Key messages**
1: Nutrition for the baby	Month 5	• Nutrition requirements for the infant from 5–6 months
		•Nutrition Introduction
		•Responsive Feeding
2: Baby's first solid foods	Month 6	•Porridge preparation and responsive feeding
3: Introducing more food to baby	Month 7	•Nutrient needs for growing infant
		•Porridge preparation with additives
4: Maintenance of breastfeeding and feeding during illness	Month 8	•Importance of continued breastfeeding
		•Feeding during illness
5: Dietary Diversity	Month 9	• Importance of diverse foods and how to incorporate them in infant diet
6: Reinforcement of 1–5	Month 12	•Recall key nutrient education on nutrient needs of baby, responsive feeding, feeding during illness and diverse food groups
		•Finger foods and self-feeding demonstration

### Population and data collection

#### Participant inclusion

**Fig. 1:**
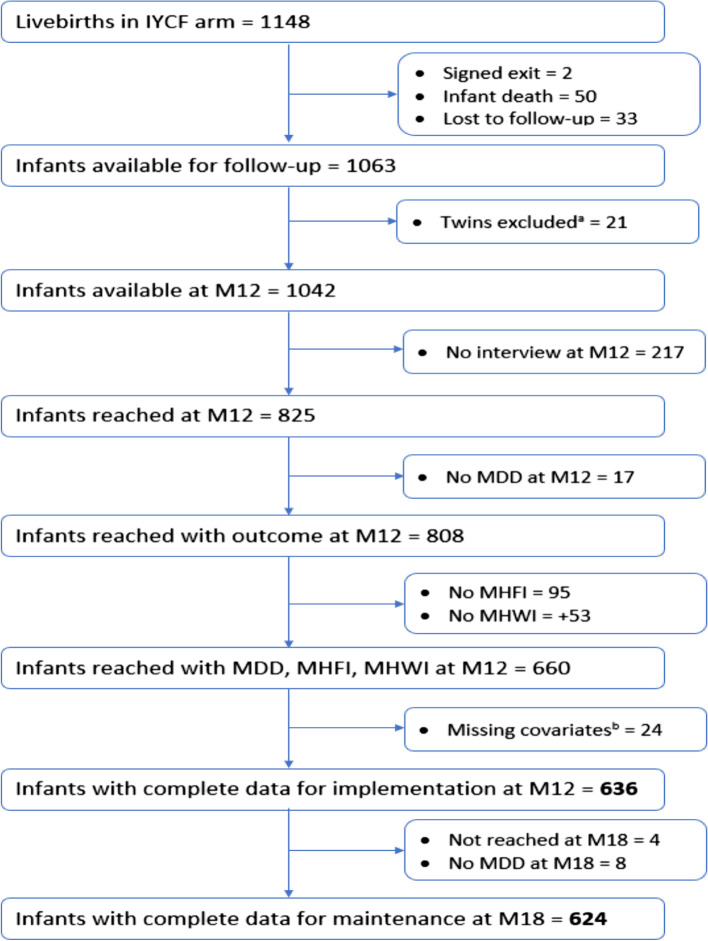
Flowchart of eligible and included mother-infant dyads. ^a^Twins were excluded since only one diet questionnaire was filled and it was not possible to determine which infant the information belonged to. ^b^Missing covariates: unknown HIV−status (*n*=1), maternal age (*n*=20), maternal education (*n*=3), all other covariates (*n*=0). MHFI= Multidimensional Household Food Insecurity. MHWI= Multidimensional Household Water Insecurity

Research nurses made home visits at multiple times to collect relevant information from mothers and infants: at baseline (during the pregnancy period) and at infant ages 1, 3, 6, 12 and 18 months. Since SHINE was household-based, the intermediate visits were conducted only when participants were available at the address where they consented. If the participants remained inaccessible after two attempts to reach out during the intermediate visits, the mother-infant dyad data were considered missing. At M18, participants were visited anywhere in Zimbabwe, even if they had moved from their initial residence. Our sample excluded infants who had died (*n* = 50), whose mothers rescinded consent (*n* = 2), and who were lost to follow-up (*n* = 33 at M12; and an additional *n* = 4 at M18). Twins were also excluded (*n* = 21 pairs) because diet was reported for only one of the infants, and it was not possible to distinguish which infant the data belonged to. Figure 1 illustrates the sample of included and excluded participants.

#### MIDD outcomes

Infant diet was assessed using the WHO infant diet assessment questionnaire, and the MIDD indicator was defined as infants who were fed at least four out of the following seven food groups: 1) grains, roots and tubers, 2) legumes and nuts, 3) dairy products, 4) flesh foods, 5) eggs, 6) vitamin-A rich fruits and vegetables, and 7) other fruits and vegetables [8]. During the post-partum period, when infants were aged M12 and M18, mothers were asked to recall what they fed their infants in the 24 h prior to the home visit interviews. The first main outcome, MIDD implementation, occurred if mothers reported feeding their infants a minimum of four food groups at M12 as described above. MIDD implementation was determined at M12 because the last nutrition education module was delivered at 9 months, with a reminder of all previous modules provided at M12 (Table 2). The second main outcome, MIDD maintenance, was categorized into four mutually exclusive groups based on the combined MIDD practices at M12 and M18 as follows: Group A = unmet MIDD at both M12 and M18, Group B = unmet MIDD at M12 only, Group C = unmet MIDD at M18 only, and Group D = met MIDD at both M12 and M18.

**Table 2:** Description of multidimensional household food insecurity and water insecurity variables

Insecurity	Dimension	Variables used for standardized scoring
MHFI	Poor food access	Preferred food (yes/ no)
		Sufficient food (yes/ no)
		Help to get food (yes/no)
		Food on credit (yes, no)
	Household shocks	Agriculture (yes/ no)
		Economic losses (yes/no)
		Death or injury (yes/no)
	Low food availability & quality	Stock of staple food (8 ordinal categories of time)
		Having a home garden (yes/no)
		Diet diversity of household (yes/ no)
MHWI	Poor water access	Distance (> 1000 m) and time (> 15 min) to drinking water and to non-drinking water
	Poor water quality	Type of drinking source, non-drinking source (improved (piped, protected ground), unimproved ground (unprotected boreholes/ wells), surface) Drinking water satisfaction (3-level)
	Low water reliability	Unavailability of water for drinking and non-drinking purposes (ever/ never in the past year)

*MHFI* Multidimensional Household Food Insecurity

*MHWI* Multidimensional Household Water Insecurity

#### FI and WI exposure variables

The Multidimensional Household Food Insecurity (MHFI) and the Multidimensional Household Water Insecurity (MHWI) measures were used as primary FI and WI exposures, respectively [46]. These measures were developed specifically for the rural Zimbabwean households and their validities were tested to ensure robustness and usefulness. In brief, factor analyses were run on groups of food- and water-related variables. The process identified multiple dimensions representing the different aspects of each FI and WI. The resulting MHFI measure was characterized by 1) poor food access, 2) household shocks, and 3) low food availability & quality, whereas MHWI was characterized by 1) poor water access, 2) poor water quality, and 3) low water reliability. Each dimension of the MHFI and MHWI was made up of aggregated groups of variables as summarized in Table 2. Standardized scores were obtained from post-estimation commands using the PCAmix package in R4.0.2 (R Foundation for Computational Statistics, Austria, Vienna).

#### Covariates

At baseline, a structured questionnaire was used to collect information on socio-demographic characteristics such as maternal age, maternal education, marital status, parity, religion, maternal employment outside the home and household size. Maternal depression, based on Edinburgh’s Postnatal Depression Scale and Mothering Self-Efficacy were collected using validated scales as described previously [47, 48]. The HIV status of women was determined using the rapid test algorithm; those who tested positive were directed to local clinics for follow-up and treatment. Socio-economic status (SES) was based on a wealth index created specifically for this population and reported in a prior publication [49]. Season at enrolment was characterized as calendar quarter during the baseline interview. Infant characteristics such as date of birth, sex, birthweight, and premature birth (gestational age < 37 weeks) were abstracted from health facility records by trained nurses. Our analyses excluded the mother-infant dyads who had incomplete information on MHFI (*n* = 95), MHWI (*n* = 148), MIDD implementation at M12 (*n* = 17), MIDD maintenance from M12 to M18 (*n* = 8) and the above-mentioned covariates (*n* = 24).

### Statistical analyses

We used descriptive statistics to summarize the characteristics of participants included in the analysis. We report medians and interquartile ranges (IQR) for the distributions of FI and WI dimensions; means and standard deviations (SD) for normally distributed variables; and frequencies and percentages for categorical variables. We fit binary logistic regression models to investigate the association of MIDD implementation at M12 (yes vs. no) with FI and WI. Since MIDD maintenance from M12 to M18 was a non-ordered categorical outcome (groups A-D), we used multinomial logistic regressions for the assessment of the relationship between MIDD maintenance and household-level FI and WI. All regression models utilized cluster-robust estimations to account for clustering of participants within study districts. All analyses were performed with all FI, and WI dimensions included simultaneously.

To identify relevant covariates for inclusion in the models, three groups of variables were defined. Group 1 included only variables that were considered theoretically critical given the exposures and population: season at baseline interview (calendar quarter), wealth index (tercile), and household location (Chirumanzu vs. Shurugwi). Group 2 included variables that are commonly controlled for in this population and in nutrition behavior change interventions: maternal HIV status (positive vs. negative), maternal age (years), maternal education (some primary, some secondary, completed secondary), maternal religion (Apostolic, other Christian, other religion), and infant sex (male vs. female). Group 3 included mothering self-efficacy (scores: 1–5), maternal depression (scores: 0–30), household size, and parity (parous, nulliparous, missing). To select model covariates, we implemented backward elimination logistic regression by forcing retention of all Group 1 and Group 2 variables and setting retention of Group 3 variables at p < 0.2. MIDD outcomes were then modelled using all variables retained. However, since none of the identified Group 3 variables changed the measures of associations by ± 10% or more, the final models were the most parsimonious models with Group 1 and Group 2 variables only, determined by AIC and BIC. The main analyses described in this section were conducted in Stata IC v.16 (StataCorp LP, College Station, TX).

### Ethics approval

The Medical Research Council of Zimbabwe (MRCZ) and the Institutional Review Board (IRB) of the Johns Hopkins Bloomberg School of Public Health reviewed and approved the SHINE trial protocol. Written informed consent was obtained from women in the local languages (Ndebele, Shona and English).

## Results

### Overall population characteristics

The available complete-case samples for the analyses of MIDD implementation at M12 and MIDD maintenance from M12 to M18 were 636 and 624, respectively. Table 3 summarizes the characteristics of these participants. Mothers were on average 26.7 (± 6.5) years old and 160.4 (± 7.2) cm tall. About half of the mothers had at least completed secondary school education, 9.1% were employed outside the home, the majority were married, 64.6% were not primiparous, and 84.3% received ≥ 10 of the 15 IYCF intervention visits. The prevalence of HIV among the mothers was ~ 15.0%. On average, infants’ birthweight was 3.10 (± 0.49) kg. About 20.3% of the infants were born premature. Less than 40.0% of the households had a latrine available. On average, households consisted of five (IQR = 3) members. The median scores of household-level FI and WI were all negative, suggesting low food and water insecurity in this sample.

**Table 3:** Description of mother-infant dyads included in analyses of minimum infant dietary diversity implementation at 12 months and maintenance from 12 to 18 months

**Characteristics**	**Implementation**	**Maintenance**
**n**	636	624
**Mothers**		
Age (years)^1^	26.7 (6.5)	26.7 (6.5)
Height (cm)^1^	160.4 (7.2)	160.4 (7.2)
Education		
Primary	108 (17)	107 (17.2)
Some secondary	231 (36.3)	224 (35.9)
Completed secondary	297 (46.7)	293 (47.0)
Married	606 (95.3)	594 (95.2)
Employed outside the home	58 (9.1)	57 (9.1)
Parity		
Parous	411 (64.6)	403 (64.6)
Nulliparous	82 (12.9)	81 (13.0)
Missing	143 (22.5)	140 (22.4)
Religion		
Apostolic	277 (43.6)	272 (43.6)
Other Christian	309 (48.6)	304 (48.7)
Other religion	50 (7.9)	48 (7.7)
HIV-negative	543 (85.4)	535 (85.7)
High fidelity of intervention	536 (84.3)	526 (84.3)
Calendar quarter at baseline		
January to March	199 (31.3)	196 (31.4)
April to June	129 (20.3)	123 (19.7)
July to September	131 (20.6)	128 (20.5)
October to December	177 (27.8)	177 (28.4)
Depression score^1a^	2.82 (3.92)	2.83 (3.94)
Mothering self-efficacy^1b^	3.98 (0.39)	3.98 (0.39)
**Households**		
SES		
Lower	155 (24.4)	152 (24.4)
Middle	241 (37.9)	238 (38.1)
Upper	240 (37.7)	234 (37.5)
Any latrine available	251 (39.5)	242 (38.8)
District		
Chirumanzu	349 (54.9)	337 (54.0)
Shurugwi	287 (45.1)	287 (46.0)
Household size^2^	5 (3)	5 (3)
**MHFI**^**2c**^		
Poor food access	-0.55 (1.27)	-0.63 (1.3)
Household shocks	-0.13 (1.24)	-0.13 (1.25)
Low food availability & quality	-0.34 (1.34)	-0.34 (1.35)
**MHWI**^**2d**^		
Poor water access	-0.59 (1.14)	-0.59 (1.12)
Poor water quality	-0.44 (2.00)	-0.44 (2.00)
Low water reliability	-0.33 (0.22)	-0.33 (0.22)
**Infants**		
Male	320 (50.3)	314 (50.3)
Preterm	129 (20.3)	127 (20.4)
Birthweight (kg)^1^	3.10 (0.49)	3.10 (0.49)
Infant MIDD outcomes*		
MIDD implementation		
No	221 (34.8)	-
Yes	415 (65.3)	-
MIDD maintenance		
Unmet at both M12 and M18	-	56 (9.0)
Unmet at M12 only	-	159 (25.5)
Unmet at M18 only	-	75 (12.0)
Met at both M12 and M18	-	334 (53.5)

All categorical variables are presented as n (%), unless otherwise specified

^1^Continuous variable, normally distributed, presented as mean (SD)

^2^Continuous variable, non−normally distributed, presented as median (IQR)

^a^Measured using the Edinburgh Postnatal Depression Scale (score: 0–30)

^b^Mothering self−efficacy measured using questions adapted from the Parenting Sense of Competence Scale and Parenting Self−Agency Measure (score: 0–5)

^c^*MHFI* Multidimensional Household Food Insecurity

^d^*MHWI* Multidimensional Household Water Insecurity

Higher scores on MHFI and MHWI indicate higher insecurity

*Based on 7 food groups: 1) grains, roots, and tubers, 2) legumes and nuts, 3) dairy products, 4) flesh foods, 5) eggs, 6) vitamin−A rich fruits and vegetables, and 7) other fruits and vegetables

### MIDD implementation at M12

Of the 636 mother-infant dyads who were reached and for whom we had complete data, 65.3% of mothers reported having implemented MIDD at M12 (Table 3).

#### FI

Low food availability & quality was associated with lower odds of MIDD implementation at M12 in the unadjusted logistic regression model (UOR = 0.77; 95% CI: 0.65, 0.91). This association remained consistent after adjusting for covariates (AOR = 0.81; 95% CI: 0.69, 0.97). The other two FI dimensions- poor food access and household shocks- were not associated with MIDD implementation at M12 (Table 4).

**Table 4:** Association between implementation of minimum infant dietary diversity (MIDD) and household-level multidimensional food insecurity and water insecurity among SHINE’s IYCF participants (*n* = 636)

**Models**	**Food Insecurity (MHFI)**			**Water Insecurity (MHWI)**		
**OR [95%CI]**	**Poor food access**	**Household shocks**	**Low food availability & quality**	**Poor water access**	**Poor water quality**	**Low water reliability**
**Unadjusted**	0.86	1.05	**0.77**	0.93	**1.24**	1.10
	[0.73, 1.01]	[0.89, 1.23]	**[0.65, 0.91]**	[0.80, 1.09]	**[1.08, 1.43]**	[0.92, 1.32]
**Adjusted**	0.91	1.00	**0.81**	0.95	**1.25**	1.08
	[0.75, 1.10]	[0.85, 1.18]	**[0.69, 0.97]**	[0.80, 1.13]	**[1.08, 1.44]**	[0.90, 1.29]

MIDD: Minimum infant dietary diversity based on 7 food groups: 1) grains, roots, and tubers, 2) legumes and nuts, 3) dairy products, 4) flesh foods, 5) eggs, 6) vitamin−A rich fruits and vegetables, and 7) other fruits and vegetables

Values in bold indicated statistically significant associations

Unadjusted model consists only of food insecurity and water insecurity measures

Adjusted model consists of food insecurity, water insecurity, SES (lower, middle, upper), season (January to March, April to June, July to September, October to December), residence district (Chirumanzu, Shurugwi), maternal age (years), maternal height (cm), maternal education (primary, some secondary, completed secondary), religion (Apostolic, other Christian, Other religion, infant sex (male, female), maternal HIV−status (positive, negative)

#### WI

Poor water quality was associated with higher odds of MIDD implementation at M12 in both unadjusted (UOR = 1.24; 95% CI: 1.08; 1.43), and in covariate-adjusted (AOR = 1.25; 95% CI: 1.08, 1.44) logistic regression models. There were no associations between MIDD implementation and poor water access, nor with low water reliability.

### MIDD maintenance from M12 to M18

Of the 624 mother-infant dyads with complete data at both M12 and M18, 53.5% of mothers reported meeting MIDD at both time points (group D). However, 9.0% of mothers were not able to meet MIDD either time points (group A); 12.0% met the MIDD at M12, but not at M18 (group C); while 25.5% met MIDD at M18, but not at M12 (group B).

#### FI

In both unadjusted (UOR = 1.36; 95% CI: 1.13, 1.64) and adjusted (AOR = 1.29; 95% CI: 1.07, 1.56) multinomial logistic regression models, low food availability & quality was associated with higher odds of mother-infant dyads belonging to group B (unmet MIDD at M12), compared to group D. There was no association between household shocks and the odds of belonging to groups A to C, compared to group D. Poor food access was associated with higher odds of belonging to group A compared to group D in the unadjusted model (UOR = 1.37; 95% CI: 1.08, 1.73), but not the multivariable-adjusted model (AOR = 1.19; 95% CI: 0.93, 1.54) (Table 5).

**Table 5:** Association between maintenance of minimum infant dietary diversity (MIDD) from M12 to M18 and household-level multidimensional food insecurity and water insecurity (*n* = 624)

**Models**^**a**^	**Food Insecurity (MHFI)**			**Water Insecurity (MHWI)**		
**OR [95%CI]**	**Poor food access**	**Household shocks**	**Low food availability & quality**	**Poor water access**	**Poor water quality**	**Low water reliability**
**Unadjusted**						
Group A: Unmet MIDD at M12 and M18	**1.37**	0.69	1.37	1.10	1.05	0.76
	**[1.08, 1.73]**	[0.52, 0.93]	[0.95, 1.96]	[0.79, 1.52]	[0.79, 1.41]	[0.48, 1.2]
Group B: Unmet MIDD at M12 only	1.06	1.04	**1.36**	1.11	**0.79**	0.97
	[0.87, 1.3]	[0.85, 1.26]	**[1.13, 1.64]**	[0.97, 1.28]	**[0.66, 0.95]**	[0.80, 1.19]
Group C: Unmet MIDD at M18 only	0.94	0.88	1.23	1.09	**1.37**	1.06
	[0.72, 1.22]	[0.70, 1.11]	[0.95, 1.60]	[0.84, 1.41]	**[1.07, 1.76]**	[0.83, 1.36]
**Adjusted**						
Group A: Unmet MIDD at M12 and M18	1.19	0.8	1.24	1.02	1.07	0.78
	[0.93, 1.54]	[0.59, 1.08]	[0.85, 1.81]	[0.69, 1.5]	[0.77, 1.48]	[0.47, 1.3]
Group B: Unmet MIDD at M12 only	1.00	1.07	**1.29**	1.09	**0.8**	0.98
	[0.79, 1.26]	[0.87, 1.31]	**[1.07, 1.56]**	[0.93, 1.28]	**[0.66, 0.96]**	[0.80, 1.21]
Group C: Unmet MIDD at M18 only	0.86	0.92	1.16	1.08	**1.39**	1.07
	[0.65, 1.14]	[0.71, 1.19]	[0.88, 1.52]	[0.83, 1.41]	**[1.08, 1.79]**	[0.84, 1.36]

MIDD: Minimum infant dietary diversity based on 7 food groups: 1) grains, roots, and tubers, 2) legumes and nuts, 3) dairy products, 4) flesh foods, 5) eggs, 6) vitamin−A rich fruits and vegetables, and 7) other fruits and vegetables

Values in bold indicated statistically significant associations

Unadjusted model consists only of food insecurity and water insecurity measures

Adjusted model consists of food insecurity, water insecurity, SES (lower, middle, upper), season (January to March, April to June, July to September, October to December), residence district (Chirumanzu, Shurugwi), maternal age (years), maternal height (cm), maternal education (primary, some secondary, completed secondary), religion (Apostolic, other Christian, Other religion, infant sex (male, female), maternal HIV−status (positive, negative)

*MHFI* Multidimensional Household Food Insecurity, *MHWI* Multidimensional Household Water Insecurity

^a^Referent group is Group D= Met MIDD at both M12 and M18

#### WI

All multinomial logistic regression models indicated that poor water access was related to higher odds of mother-infant dyads belonging to MIDD maintenance groups A to C, compared to group D. However, these associations were not statistically significant. The associations with poor water quality were inconsistent across MIDD maintenance groups. Compared to group D, poor water quality was associated with lower odds of belonging to group B (AOR = 0.80; 95% CI: 0.66, 0.96), but higher odds of belonging to group C (AOR = 1.39; 95% CI: 1.08, 1.79). There was no association between MIDD maintenance and low water reliability.

## Discussion

In this study, we investigated the association of MIDD implementation and MIDD maintenance with household FI and WI in rural Zimbabwe, among mothers who received a nutrition intervention consisting of infant feeding education and a daily supply of 20 g/d of lipid-based infant nutrient supplement.

### FI, MIDD implementation and MIDD maintenance

Of the three dimensions of FI investigated, low food availability & quality, but not poor food access, nor household shocks, was associated with MIDD implementation and maintenance. Negative associations between FI and various infant feeding practices have been reported in Ghana [29], Ethiopia [50–52], Bangladesh [32, 33, 35, 53], India [27], and Kenya [36]. Interestingly, in Mongolia, household FI was not associated with MIDD [54]. Our results may not be directly comparable to these previous studies, however, due to lack of equivalency among FI metrics. FI assessment in the above studies was based on a single score that aggregates multiple aspects of FI, such as the Household Food Insecurity Access Scale (HFIAS) [27, 32, 36, 50, 51, 54], Food Consumption Score (FCS) [33], Household Hunger Scale (HHS) [29], or country-specific food insecurity access scale [33, 35]. In contrast, our FI assessment was based on three measures that quantified different dimensions of FI (Table 2). Food availability & quality was indicated by the presence of staple food reserves, a home garden and at least one household member having eaten diverse foods. Therefore, lacking in these aspects constitutes a barrier for MIDD implementation and maintenance in this population. It should also be noted that the finding with the dimension low food availability and quality may be an artifact of the way that dimension was created. For instance, when the variable household dietary diversity was removed from that dimension, the associations with MIDD implementation and maintenance were no longer significant.

Most of the existing studies are cross-sectional and rarely assess the contribution of FI to MIDD implementation and maintenance within a nutrition intervention. A prospective cohort in rural Bangladesh, without CF intervention, found that infants from households with higher baseline food security scores were more likely to meet the MIDD at 9 months (OR = 3.6; 95% CI: 2.2, 4.6) [33]. To our knowledge, only one longitudinal study reported this association within a nutrition education intervention [36]. The study, implemented in urban Kenya, found that infants from food secure households were 80% more likely to achieve MIDD-8 between 6 to 23 months of age than those living in food insecure households.

It is also plausible that education was the major barrier to MIDD in this Zimbabwean population, rather than poor food access and household shocks. Indeed, the same analysis performed with mother-infant dyads from SHINE’s SOC arm did not identify any association between FI dimensions and MIDD implementation (Table S1). As has been shown in Malawi, nutrition education for CF behavior change even among food insecure households improves infant dietary diversity [55]. For MIDD maintenance, infants in the SOC arm were more likely to be in group B with poorer food access (Table S2). Both our main and sensitivity analyses showed that low food availability & quality was associated with higher likelihood of infants belonging to the poor MIDD maintenance group B, compared to the adequate MIDD maintenance group D. These results suggest that the drivers of FI are differentially important for MIDD implementation and maintenance, even among those who receive nutrition education on CF.

We did not observe an association with household shocks, a measure of food supply stability in the year prior to the interview. It is possible that the households recovered from the shocks by the time their infants were 12–18 months of age or have found ways to cope with FI that allows adequate infant feeding practices. In post-hoc analyses, household shocks were associated with higher odds of coping strategies such as hunting and gathering wild foods (OR = 1.71; 95% CI: 1.19, 2.43) and harvesting green maize for food (OR = 1.56; 95% CI: 1.07, 2.25). These behaviors are consistent with households that experience food insecurity and adopt certain behaviors to minimize its negative consequences [56].

### WI, MIDD implementation and MIDD maintenance

Contrary to our hypothesis, poor water quality, but no other dimension of WI, was associated with higher odds of MIDD implementation. Poor water quality was defined by three variables: the main type of water source used by the household for drinking and consumption, for non-drinking purposes, and the level of satisfaction with the drinking water source (Table 2). This could be explained by the categorization of water sources. For example, a recent study in India also used primary drinking water source as a measure of household water access instead of water quality [37]. The study reported that infants from households with sub-optimal water access were 2% less likely to meet the MIDD, compared to those with optimal water access. In this case, sub-optimal water sources included water piped to neighbor, public tap, tube well/ borehole, protected well/ spring, dug well, unprotected well/ spring, river/ dam, rainwater, and others. In our study, all piped and protected water sources (piped into dwelling, piped on-plot, piped off-plot, protected boreholes/ wells/ streams, and bottled) were considered of good quality, while all ground (unprotected boreholes, wells, streams) and surface water (rivers, riverbanks, shallow holes) were considered unimproved sources. These differences reflect the differences in WI definitions and water situations between rural Zimbabwe and India.

Additionally, 65% of rural Zimbabwean households had access to improved drinking water, but an equal proportion used unimproved ground and surface water for non-drinking purposes. Based on qualitative reports of mothers’ perception that water of poor quality may carry disease-causing agents or require more work to make the water safe [40], the non-drinking water sources were unlikely used for food preparation. However, the high percentage of households using unimproved water for other purposes may be driving the higher poor water quality scores, in turn inflating our observed associations.

Post hoc analyses revealed other plausible explanations. First, unimproved ground water for drinking purposes were closer (464 m vs. 608 m; *p* < 0.01), and therefore more rapidly accessible than improved sources (11 vs 13 min; *p* = 0.09). Mothers in rural areas who are already burdened by household chores, child-caring activities and other responsibilities may choose these unimproved low-quality sources to minimize the time and effort associated with water collection [39]. Second, poorer water quality was associated with higher likelihood of households engaging in water treatment, such as boiling and using chlorine or bleach (OR = 1.89; 95% CI: 1.45, 2.47). Thus, poor initial water quality did not necessarily preclude water use for consumption and food preparation. Third, non-drinking water sources were linked to the households’ agricultural productivity. Households with unimproved water as non-drinking source were more likely to engage in irrigation (OR = 1.73; 95% CI: 1.01, 1.96). Among farming-majority rural households, like our population, water is essential to enhance agricultural productivity for improved nutrition and health [57–59].

The associations of poor water quality on MIDD maintenance were inconsistent. First, poor water quality was associated with higher odds of being in group C (unmet MIDD at M18 only), which supports our hypothesis. Second, contrary to our hypothesis, poor water quality was associated with lower odds of being in group B (unmet MIDD at M12 only). This was also true among households that received at least 10 of the 15 IYCF visits. Households tend to vary their water sources by season, and are more likely to use low quality sources during the dry season to meet their needs [60]. Thus, our results for MIDD maintenance may be an artifact of seasonality and climate situations that varied between baseline, and infant ages of M12 and M18.

### Strengths and limitations

To our knowledge, this study is the first study to integrate FI and WI simultaneously in associations with infant feeding practices. This study is also the first to test these associations with different critical dimensions of FI and WI. The Food and Agriculture Organization (FAO) [22] and the Joint Monitoring Programme for Water Supply, Sanitation and Hygiene (JMP) [61] have both identified the need for separate indicators of the dimensions of FI and WI to better target relevant interventions. So far, nutrition-sensitive, and nutrition-specific interventions have mostly focused on maximizing one aspect of food or water (e.g., food supplementation, fortification or drinking water treatment). By identifying the multidimensional roles of WI and FI in shaping CF, our analyses suggest other potential targets for nutrition policies and interventions.

Understanding the context that determines infant diets and caregiver feeding practices is also critical for improving CF interventions [62, 63]. A small study comparing Zimbabwe and Tanzania reported that to improve infant feeding, Zimbabwean infants needed fortified food supplements and improved agriculture, while Tanzanian infants needed only fortified food supplements [64]. In Malawi, context-specific behavior-change intervention allowed improved hygiene practices during complementary food preparation [65]. Our MHFI and MHWI measures further allow us to explore caregivers’ feeding behaviors based on specific food and water constraints in rural resource-poor settings. Such indicators may complement existing FI and WI metrics during decision making on resource allocation and research coordination with combined food and water considerations.

This paper uses data obtained from a community-based randomized controlled trial within a vulnerable population of mother-infant dyads in an LMIC. Although the associations reported here were not a core objective of SHINE, longitudinal data collection consisted of multiple follow-up times with the FI, and WI exposures assessed prior to the start of CF. These strengthened our ability to avoid reverse causation bias, and to investigate the MIDD practice and maintenance over time.

One limitation is that the MHFI and MHWI measures were calculated at baseline when the mothers were pregnant. Whereas we assumed that all dimensions of household FI and WI were valid for the post-partum period, associations may change with seasonal variations in FI and WI [58, 66, 67]. To account for this, we adjusted for seasonality of the baseline interview in our main models, and additionally conducted sensitivity analyses by restricting participants interviewed during the dry season (April to October), during the season of plenty (April to December), and with similar interview season at baseline and endpoint. These yielded findings consistent with the main models, although some associations, such as water access, became statistically significant (Tables S1 and S2). Future studies will benefit from lagged seasonal models, or from using FI and WI as time-varying exposures.

Second, our sample size did not provide sufficient power to investigate interactions or mediations between FI and WI on MIDD implementation or maintenance. Health studies have previously reported synergistic effects of FI and WI on maternal depression [68], and mediating effect of FI in the association between WI and anxiety or depression [69]. Similar relationships are possible with infant feeding practices since FI and WI compound each other in a number of ways [17].

Third, the study outcomes were based on maternal recall of infant feeding. Since the mothers received CF education meant to change their feeding knowledge and behaviors [70], their reported feeding practices may be affected by social desirability bias, thus attenuating the true associations. We also assumed that the single 24HR at M12 and M18 represent the usual daily intakes of infants during that period. Ideally, three days of 24HR provide more accurate estimation of habitual diet [71].

Fourth, we only had information on MIDD, whereas other indicators such as minimum meal frequency (MMF) and minimum adequate diet (MAD) have also been recommended [72]. Furthermore, we have used MIDD as a binary variable, which may have compromised some precision in the data. Nevertheless, during the CF window of 6–23 months, MIDD is a good proxy for nutrient adequacy in infant populations [8]. In ordinal logistic regression with diet diversity score at M12, results for FI and WI were consistent with the main findings (Table S1).

Finally, 39% of the eligible sample was excluded due to missed M12 interviews and missing relevant variables. Compared to those who were included, a higher percentage of those who were excluded from our main analyses were in the lower SES, a lower percentage received at least 10 out of the 15 IYCF intervention modules, and the mothers were younger. Inverse-probability weighting accounting for these missing data yielded findings consistent with the main models (Tables S1 and S2).

## Conclusion

Among rural Zimbabwean mothers who received a CF nutrition intervention, both FI and WI were important for the implementation and maintenance of a minimum diverse diet for infants. Specifically, low food availability & quality was identified as a barrier to implementation at M12 and with maintenance at M18. Although poor water quality was associated with poor MIDD implementation, the associations were mixed for MIDD maintenance. Additional studies on WI and its dimensions in relation to infant feeding behaviors are required to confirm consistency and direction of associations, also accounting for socio-environmental factors.

## Supplementary Information

**Additional file 1: Table S1. **Sensitivity analyses of the association between implementation of minimum infant dietary diversity at 12 months and household-level multidimensional food insecurity and water insecurity. **Table S2.** Sensitivity analyses of associations of minimum infant dietary diversity maintenance from 12 to 18 months and multidimensional household-level food insecurity and water insecurity.

## Data Availability

The datasets generated and/or analysed during the current study are not publicly available due participant privacy, but are available from the corresponding author on reasonable request, or from the Zvitambo Institute for Maternal and Child Health Research.

## Abbreviations

FI: Food Insecurity
MHFI: Multidimensional Household Food Insecurity measure
MHWI: Multidimensional Household Water Insecurity measure
MIDD: Minimum Infant Dietary Diversity
SHINE: Sanitation, Hygiene, and Infant Nutrition Efficacy trial
WI: Water Insecurity
