# Supplementary material for: Implementation and maintenance of infant dietary diversity in Zimbabwe: contribution of food and water insecurity
Source: BMC Nutr. 2022 Nov 18;8:136. doi: 10.1186/s40795-022-00622-8 (PMC9673371; doi:10.1186/s40795-022-00622-8)
Supplement: Supplementary file 1 — Additional file 1: Table S1. Sensitivity analyses of the association between implementation of minimum infant dietary diversity at 12 months and household-level multidimensional food insecurity and water insecurity. Table S2. Sensitivity analyses of associations of minimum infant dietary diversity maintenance from 12 to 18 months and multidimensional household-level food insecurity and water insecurity. [file 40795_2022_622_MOESM1_ESM.docx]

Table S1: Sensitivity analyses of the association between implementation of minimum infant dietary diversity at 12 months and household-level multidimensional food insecurity and water insecurity

| OR  [95% CI] | MIDD | Food insecurity (MHFI) | | | Water Insecurity (MHWI) | | |
| --- | --- | --- | --- | --- | --- | --- | --- |
|  | n/ N | Poor food access | Household shocks | Low food availability & quality | Poor water access | Poor water quality | Low water reliability |
| MIDD-8 | 406/ 636 | 0.92 | 1.02 | 0.83 | 0.93 | 1.2 | 1.07 |
|  |  | [0.77, 1.10] | [0.87, 1.20] | [0.70, 0.98] | [0.77, 1.11] | [1.04, 1.4] | [0.89, 1.28] |
| High fidelity of IYCF intervention only^a^ | 346/ 536 | 0.91 | 1.02 | 0.81 | 0.96 | 1.24 | 1.16 |
|  |  | [0.73, 1.14] | [0.84, 1.22] | [0.69, 0.97] | [0.8, 1.13] | [1.09, 1.43] | [0.98, 1.37] |
| HIV-negative mothers only | 359/ 543 | 0.89 | 1.04 | 0.82 | 0.95 | 1.18 | 1.08 |
|  |  | [0.72, 1.10] | [0.87, 1.25] | [0.68, 1.00] | [0.79, 1.15] | [1.01, 1.39] | [0.89, 1.31] |
| Season of plenty at baseline^b^ | 285/ 437 | 0.86 | 0.96 | 0.74 | 0.84 | 1.27 | 1.14 |
|  |  | [0.68, 1.07] | [0.80, 1.15] | [0.59, 0.93] | [0.69, 1.02] | [1.07, 1.51] | [0.92, 1.41] |
| Season of plenty at M12^b^ | 309/ 462 | 0.88 | 1.07 | 0.78 | 1.06 | 1.35 | 1.07 |
|  |  | [0.69, 1.12] | [0.88, 1.29] | [0.62, 0.99] | [0.86, 1.32] | [1.10, 1.65] | [0.87, 1.32] |
| Dry season at baseline^c^ | 212/ 323 | 0.89 | 0.88 | 0.62 | 0.75 | 1.35 | 1.18 |
|  |  | [0.71, 1.12] | [0.70, 1.11] | [0.47, 0.83] | [0.59, 0.95] | [1.10, 1.66] | [0.88, 1.58] |
| Dry season at M12^c^ | 259/ 389 | 0.87 | 1.08 | 0.86 | 1.12 | 1.34 | 1.08 |
|  |  | [0.66, 1.15] | [0.86, 1.35] | [0.66, 1.13] | [0.86, 1.45] | [1.08, 1.65] | [0.86, 1.35] |
| MIDD scores^d^ | 636 | 0.91 | 1 | 0.81 | 0.95 | 1.24 | 1.08 |
|  |  | [0.75, 1.10] | [0.85, 1.18] | [0.68, 0.97] | [0.80, 1.13] | [1.08, 1.44] | [0.90, 1.29] |
| Inverse probability weighted model | 415/ 632 | 0.9 | 0.99 | 0.80 | 0.96 | 1.24 | 1.05 |
|  |  | [0.75, 1.08] | [0.84, 1.17] | [0.66, 0.97] | [0.79, 1.16] | [1.04, 1.47] | [0.86, 1.29] |
| Standard of care MIDD-7 | 322/ 596 | 1.08 | 1.08 | 0.95 | 0.86 | 0.93 | 0.95 |
|  |  | [0.93, 1.25] | [0.93, 1.25] | [0.79, 1.15] | [0.68, 1.08] | [0.80, 1.08] | [0.79, 1.13] |

n/ N= number of mothers who implemented the minimum infant dietary diversity/ total number of participating mothers

MHFI= Multidimensional Household Food Insecurity

MHWI= Multidimensional Household Water Insecurity

Standard of care refers to the control group of the SHINE trial

All analyses are carried out on IYCF participants unless otherwise specified

All analyses are based on 7 food groups unless otherwise stated

MIDD-7: (1) grains, roots and tubers, 2) legumes and nuts, 3) dairy products, 4) flesh foods, 5) eggs, 6) vitamin-A rich fruits and vegetables, and 7) other fruits and vegetables (MIDD=4/7)

MIDD-8: (1) grains, roots and tubers, 2) legumes and nuts, 3) dairy products, 4) flesh foods, 5) eggs, 6) vitamin-A rich fruits and vegetables, 7) other fruits and vegetables, 8) breastmilk (MIDD=5/8)

All analyses were multivariable binary logistic regressions unless otherwise stated

Unless otherwise stated, all models were adjusted for food insecurity, water insecurity, SES (lower, middle, upper), season (January to March, April to June, July to September, October to December), residence district (Chirumanzu, Shurugwi), maternal age (years), maternal height (cm), maternal education (primary, some secondary, completed secondary), religion (Apostolic, other Christian, Other religion, infant sex (male, female), maternal HIV-status (positive, negative)

a High fidelity: at least 10 education modules received out of 15

b Season of plenty: April to December

c Dry season: April to October

d Ordinal logistic regression with seven categories based on number of food groups consumed by infants (range: 1-7)

Table S2: Sensitivity analyses of associations of minimum infant dietary diversity maintenance from 12 to 18 months and multidimensional household-level food insecurity and water insecurity

| Models | MIDD | Food insecurity (MHFI) | | | Water Insecurity (MHWI) | | |
| --- | --- | --- | --- | --- | --- | --- | --- |
| OR [95%CI] | n | Poor food access | Household shocks | Low availability & quality | Poor water access | Poor water quality | Low water reliability |
| MIDD-8 | 624 |  |  |  |  |  |  |
| A | 83 | 1.15 | 0.79 | 1.29 | 1 | 0.96 | 0.89 |
|  |  | [0.92, 1.44] | [0.59, 1.06] | [1.01, 1.64] | [0.73, 1.37] | [0.76, 1.2] | [0.62, 1.27] |
| B | 140 | 0.94 | 1.11 | 1.33 | 1.13 | 0.84 | 0.97 |
|  |  | [0.76, 1.15] | [0.89, 1.39] | [1.06, 1.66] | [0.94, 1.36] | [0.68, 1.03] | [0.78, 1.2] |
| C | 289 | 0.78 | 0.97 | 1.19 | 1.07 | 1.22 | 1.01 |
|  |  | [0.57, 1.08] | [0.76, 1.23] | [0.89, 1.59] | [0.86, 1.34] | [0.96, 1.54] | [0.81, 1.26] |
| High fidelity of IYCF intervention only^a^ | 526 |  |  |  |  |  |  |
| A | 50 | 1.07 | 0.83 | 1.26 | 0.93 | 1.02 | 0.6 |
|  |  | [0.81, 1.41] | [0.6, 1.17] | [0.83, 1.9] | [0.67, 1.3] | [0.75, 1.38] | [0.33, 1.1] |
| B | 134 | 1.01 | 1.02 | 1.29 | 1.11 | 0.81 | 0.95 |
|  |  | [0.78, 1.31] | [0.82, 1.27] | [1.06, 1.56] | [0.92, 1.33] | [0.68, 0.98] | [0.79, 1.15] |
| C | 69 | 0.81 | 0.86 | 1.19 | 1.06 | 1.38 | 1.13 |
|  |  | [0.61, 1.08] | [0.66, 1.11] | [0.87, 1.61] | [0.8, 1.39] | [1.06, 1.79] | [0.87, 1.47] |
| HIV-negative mothers only | 535 |  |  |  |  |  |  |
| A | 445 | 1.29 | 0.82 | 1.27 | 1.08 | 1.16 | 0.93 |
|  |  | [0.94, 1.77] | [0.58, 1.16] | [0.82, 1.97] | [0.71, 1.66] | [0.8, 1.68] | [0.58, 1.48] |
| B | 134 | 0.97 | 1.02 | 1.25 | 1.07 | 0.83 | 0.95 |
|  |  | [0.76, 1.24] | [0.81, 1.27] | [1.01, 1.53] | [0.9, 1.28] | [0.69, 1.01] | [0.75, 1.21] |
| C | 70 | 0.75 | 0.94 | 1.12 | 1.07 | 1.34 | 1.13 |
|  |  | [0.54, 1.04] | [0.71, 1.25] | [0.85, 1.48] | [0.8, 1.43] | [1.02, 1.75] | [0.88, 1.44] |
| Season of plenty at baseline^b^ | 428 |  |  |  |  |  |  |
| A | 43 | 1.15 | 0.94 | 1.31 | 1.18 | 0.98 | 0.89 |
|  |  | [0.88, 1.50] | [0.69, 1.29] | [0.93, 1.85] | [0.83, 1.67] | [0.75, 1.29] | [0.60, 1.31] |
| B | 106 | 0.98 | 1.18 | 1.40 | 1.31 | 0.8 | 0.85 |
|  |  | [0.77, 1.25] | [0.90, 1.54] | [1.13, 1.74] | [1.06, 1.62] | [0.61, 1.03] | [0.67, 1.08] |
| C | 48 | 0.86 | 1.08 | 1.25 | 1.17 | 1.24 | 0.91 |
|  |  | [0.61, 1.21] | [0.79, 1.47] | [0.91, 1.71] | [0.90, 1.50] | [0.94, 1.64] | [0.71, 1.17] |
| Dry season at baseline^c^ | 314 |  |  |  |  |  |  |
| A | 29 | 1.12 | 1.06 | 1.58 | 1.36 | 0.92 | 0.79 |
|  |  | [0.85, 1.49] | [0.71, 1.59] | [1.03, 2.41] | [0.91, 2.03] | [0.65, 1.30] | [0.47, 1.35] |
| B | 79 | 0.94 | 1.3 | 1.60 | 1.54 | 0.85 | 0.8 |
|  |  | [0.70, 1.25] | [0.99, 1.70] | [1.19, 2.14] | [1.22, 1.95] | [0.62, 1.15] | [0.59, 1.09] |
| C | 37 | 0.78 | 1.1 | 1.04 | 1.17 | 1.54 | 0.85 |
|  |  | [0.53, 1.14] | [0.77, 1.55] | [0.72, 1.49] | [0.85, 1.60] | [1.10, 2.16] | [0.61, 1.18] |
| Same season at baseline and M18 | 333 |  |  |  |  |  |  |
| A | 31 | 1.05 | 1.05 | 1.22 | 1.19 | 1.04 | 0.29 |
|  |  | [0.71, 1.54] | [0.72, 1.54] | [0.75, 1.96] | [0.74, 1.91] | [0.71, 1.53] | [0.06, 1.33] |
| B | 94 | 0.82 | 1.02 | 1.29 | 1.04 | 0.71 | 1.04 |
|  |  | [0.56, 1.19] | [0.81, 1.29] | [0.99, 1.67] | [0.8, 1.34] | [0.53, 0.95] | [0.76, 1.41] |
| C | 43 | 0.64 | 1 | 0.91 | 1.15 | 1.35 | 1.05 |
|  |  | [0.42, 0.96] | [0.67, 1.5] | [0.64, 1.3] | [0.74, 1.78] | [0.89, 2.06] | [0.78, 1.41] |
| Inverse probability weighted model | | | | | | | |
| A |  | 1.33 | 0.78 | 1.19 | 1.05 | 1.15 | 0.93 |
|  |  | [1.02, 1.72] | [0.58, 1.06] | [0.80, 1.77] | [0.69, 1.61] | [0.81, 1.65] | [0.57, 1.50] |
| B |  | 1.00 | 1.09 | 1.33 | 1.08 | 0.78 | 0.98 |
|  |  | [0.80, 1.25] | [0.88, 1.36] | [1.07, 1.64] | [0.91, 1.27] | [0.64, 0.97] | [0.78, 1.23] |
| C |  | 0.89 | 0.95 | 1.15 | 1.09 | 1.40 | 1.05 |
|  |  | [0.65, 1.22] | [0.72, 1.27] | [0.88, 1.51] | [0.82, 1.44] | [1.07, 1.82] | [0.82, 1.33] |
| Standard of care MIDD-7 | 590 |  |  |  |  |  |  |
| A | 90 | 1.16 | 0.81 | 0.85 | 1.11 | 1.04 | 1.14 |
|  |  | [0.88, 1.53] | [0.64, 1.03] | [0.68, 1.06] | [0.77, 1.58] | [0.77, 1.4] | [0.89, 1.46] |
| B | 181 | 1.27 | 1.05 | 1.08 | 1.25 | 1.13 | 1.07 |
|  |  | [1.02, 1.58] | [0.86, 1.29] | [0.84, 1.38] | [0.98, 1.6] | [0.94, 1.35] | [0.85, 1.35] |
| C | 76 | 1.24 | 1.17 | 0.83 | 1.06 | 1.03 | 1.05 |
|  |  | [0.96, 1.61] | [0.94, 1.46] | [0.6, 1.14] | [0.74, 1.52] | [0.8, 1.32] | [0.8, 1.4] |

n= number of participants in each maintenance group

MHFI= Multidimensional Household Food Insecurity

MHWI= Multidimensional Household Water Insecurity

MIDD-7: (1) grains, roots and tubers, 2) legumes and nuts, 3) dairy products, 4) flesh foods, 5) eggs, 6) vitamin-A rich fruits and vegetables, and 7) other fruits and vegetables (MIDD=4/7)

MIDD-8: (1) grains, roots and tubers, 2) legumes and nuts, 3) dairy products, 4) flesh foods, 5) eggs, 6) vitamin-A rich fruits and vegetables, 7) other fruits and vegetables, 8) breastmilk (MIDD=5/8)

MIDD maintenance groups: A=Unmet MIDD at M12 and M18; B=Unmet at M12 only; C=Unmet at M18; D= Met MDD at both M12 and M18

Standard of care refers to the control group of the SHINE trial

All analyses are based on 7 food groups unless otherwise stated

All comparisons are in reference to group D (met MIDD at both M12 and M18)

All analyses are carried out on IYCF participants unless otherwise specified

All analyses were multivariable multinomial logistic regressions unless otherwise stated

Unless otherwise stated, all models were adjusted for food insecurity, water insecurity, SES (lower, middle, upper), season (January to March, April to June, July to September, October to December), residence district (Chirumanzu, Shurugwi), maternal age (years), maternal height (cm), maternal education (primary, some secondary, completed secondary), religion (Apostolic, other Christian, Other religion, infant sex (male, female), maternal HIV-status (positive, negative)

^a^ High fidelity: at least 10 education modules received out of 15

^b^ Season of plenty: April to December

^c^ Dry season: April to October
